# Population dynamics of sympatric *Phortica* spp. and first record of stable presence of *Phortica oldenbergi* in a *Thelazia callipaeda*-endemic area of Italy

**DOI:** 10.1186/s13071-024-06526-9

**Published:** 2024-11-06

**Authors:** Ilaria Bernardini, Cristiana Poggi, Daniele Porretta, Jan Máca, Eleonora Perugini, Sara Manzi, Simona Gabrielli, Verena Pichler, Maria Stefania Latrofa, Josephus Fourie, Riccardo Paolo Lia, Frédéric Beugnet, Domenico Otranto, Marco Pombi

**Affiliations:** 1 Dipartimento di Sanità Pubblica e Malattie Infettive, Sapienza Università di Roma, Rome, Italy; 2 Dipartimento di Malattie Infettive, Istituto Superiore di Sanità, Rome, Italy; 3 Dipartimento di Biologia Ambientale, Sapienza Università di Roma, Rome, Italy; 4 Czech Entomological Society, Prague, Czech Republic; 5 Dipartimento di Medicina Veterinaria, Università degli Studi di Bari, Valenzano, Bari, Italy; 6 Boehringer-Ingelheim Animal Health, Lyon, France; 7 ClinVet International (Pty) Ltd, Bloemfontein, South Africa; 8 Department of Veterinary Clinical Sciences, City University of Hong Kong, Kowloon Tong, Hong Kong, China

**Keywords:** Phortica, Sympatry, Eyeworm, Thelazia, Zoonosis, Lachryphagy, Wolbachia, Vector-borne disease

## Abstract

**Background:**

Five species of the *Phortica* genus (Diptera: Drosophilidae) are known in Europe and the Middle East. Among these, *Phortica variegata* and *Phortica okadai* are better known for their role as vectors of the zoonotic eyeworm *Thelazia callipaeda*. Other species, such as *Phortica semivirgo* and *Phortica oldenbergi*, have been studied less. Given the paucity of data about these *Phortica* spp. vectors, we explored the population dynamics and ecology of *Phortica* spp. in an area highly endemic for *T*. *callipeada* (Manziana, Rome, Central Italy).

**Methods:**

*Phortica* spp. flies were collected over a 3-year period (2018–2020) during their active season (April–October) with a sweep net while hovering around fermenting fruits or a human operator acting as baits. Collected flies were morphologically identified and tested for a *T*. *callipeada* infection and for the presence of *Wolbachia*, by polymerase chain reaction (PCR). Population dynamics of species collected was associated to environmental drivers through generalized additive models.

**Results:**

Of the 5564 flies collected, 90.8% were *P*. *variegata*, 9.1% were *P*. *oldenbergi*, 0.05% were *P*. *semivirgo*, and one specimen was *P*. *okadai*. Only *P*. *variegata* scored molecularly infected with *T*. *callipeada* throughout the 3-year sampling period (1.8%). *Phortica oldenbergi*, observed consistently during the entire sampling period, exhibited a marked preference for fruit traps, contrasting with the lachryphagous activity of *P*. *variegata*. Analysis of environmental drivers of *P*. *oldenbergi* and *P*. *variegata* population dynamics indicated temperature, wind speed, and pressure as significant factors. In addition, *Wolbachia pipientis* endosymbiont was detected in *P*. *oldenbergi* and *P*. *okadai*.

**Conclusions:**

For the first time, this study analysed several ecological aspects of *Phortica* species coexisting in a *T*. *callipeada* endemic area, highlighting different behaviors in the same environment and their vectorial role. Notably, this is also the first report of the presence of *P*. *oldenbergi* in Italy and *P*. *okadai* in Europe, underscoring the importance of extensive sampling for detecting potential vectors and alien species with direct implications for vector-borne disease epidemiology.

**Graphical Abstract:**

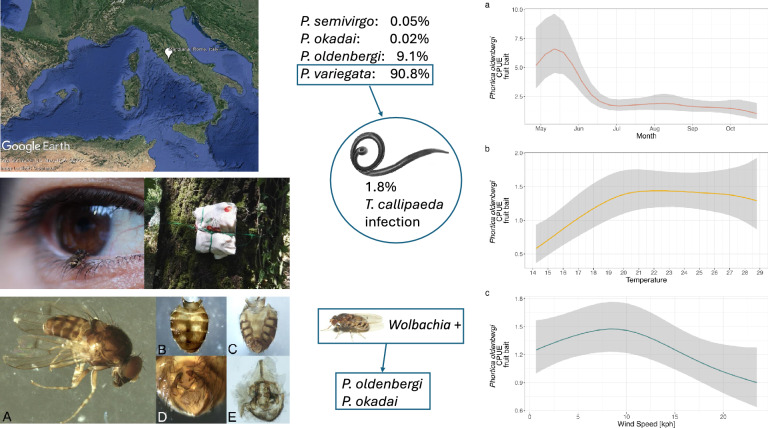

**Supplementary Information:**

The online version contains supplementary material available at 10.1186/s13071-024-06526-9.

## Background

The genus *Phortica* Schiner (1862) (Diptera, Drosophilidae, Steganinae) counts almost 160 species distributed all over the world [1]. Nearly two-thirds of these are described in East Asia, especially in southern China. The importance of this taxonomic group in medical and veterinary entomology is rising owing to its role as vector for the eyeworm *Thelazia callipaeda* (Railliet and Henry, 1910) (Spirurida, Thelaziidae), which causes infection in many mammal species [2–4]. Indeed, while feeding on animal secretions, lachryphagous males of some *Phortica* species transmit this eyeworm to definitive hosts such as dogs, cats, and rabbits, wildlife (e.g., foxes, hares, wolfs, mustelids, and bears), and occasionally humans [5–15]. Adult worms develop in the conjunctival sac of mammal hosts causing mild to severe outcomes, such as conjunctivitis, keratitis, corneal ulcer, and potentially blindness [8, 12, 16–18]. Thelaziosis has been considered endemic in Asia, and emerging across Europe, [16, 18] and in USA [19]. Major natural vectors are *Phortica okadai* (Máca,1977) and *Phortica variegata* (Fàllen,1823), while *Phortica kappa* (Máca, 1977) and *Phortica magna* (Okada, 1960) are only recently described as vectors [15]. Moreover, *Phortica oldenbergi* (Duda, 1921) has been described as a competent vector of *T*. *callipeada* in laboratory conditions [20].

Concerning the distribution of *Phortica* spp. in the European continent, *P*. *variegata* and *P*. *semivirgo* (Máca,1977) are widely spread [21–23], while other species have been sporadically reported in few countries: *Phortica erinacea* (Máca,1977) in Bulgaria/Greece, *Phortica goetzi* (Máca,1987) in Turkey, and *P*. *oldenbergi* in Germany and Spain at the beginning of the twentieth century [24–26]. In particular, only three specimens *P*. *oldenbergi* were collected in Germany at the beginning of the twentieth century [24, 25] while the more recent report in Spain is based upon morphological identification of the females not supported by molecular data [26].

Excepting *P*. *variegata*, little is known about the distribution, ecology, and biology of *T*. *callipeada* vectors in Europe [21]. For this vector, some information on its life history traits is available, and it has been reconstructed from laboratory breeding [27, 28] and field sampling. This species is associated with oak forests (*Quercus* spp.) [9, 29, 30] where flies are found active from April–May to October–November [9, 30]. This is followed by an overwintering period of adults for both *P*. *variegata* and *P*. *semivirgo* [3, 31], with some specimens collected in caves during winter [9, 32, 33]. However, knowledge on the ecology of *P*. *variegata* is limited, particularly with regard to larval ecology.

Moreover, no information is available about the presence and ecological contribution of endosymbionts in *Phortica* species, despite evidence on the absence of *Wolbachia pipientis* (Hertig, 1936) in *T*. *callipeada* [34]. *Wolbachia* is an obligate intracellular maternally transmitted gram-negative genus establishing a spectrum of symbiotic relationships, ranging from parasitism to obligatory mutualism. Up to 50% of terrestrial arthropod species, and several onchocercid nematodes harbor this endosymbiont in nature, including several Drosophilidae [35].

In this study, we investigated the presence and the population dynamics of *Phortica* spp*.* in the ecological setting of Central Italy, Manziana, an area known for the presence of these flies and endemic to *T*. *callipeada* [30]. Here we report, for the first time, the co-occurrence of four *Phortica* species, detailed data about their ecology and phenology, as well as the first record of a stable population of *P*. *oldenbergi* in Italy. Data on the potential expansion in the distribution of these *Phortica* spp. are also discussed in light of the risk they may represent as competent vectors of *T*. *callipeada*.

## Methods

### *Phortica* spp. sample collection and morphological identification

Longitudinal samplings were performed in the oak forest of Manziana (Rome, Italy, 42°07′10.8″N—12°06′57.6″E) between the months of April and October from 2018 to 2020.

*Phortica* spp. flies were collected with a sweep net while hovering around two baits: a fermenting fruit bait for flies showing a frugivorous trophic behavior, or a human operator acting as bait for lachryphagous flies [8]. Specimens were killed soon after being collected and stored in 70% ethanol in the field. After collection, all specimens were morphologically identified by species and sex [24, 36]. In case of doubtful identification (*N* = 95), a molecular confirmation was carried out with conventional polymerase chain reaction (PCR) and sequencing.

### *Phortica* spp. molecular identification

Genomic DNA was isolated from single specimens using DNAzol DNA extraction protocol (MCR, Inc., Cincinnati Ohio) and then amplified with primers UEA7 (5′- TACAGTTGGAATAGACGTTGATAC-3′) and UEA10 (5′-TCCAATGCACTAATCTGCCATATTA-3′) to target the mitochondrial cytochrome oxidase subunit I gene (*cox1*) [37]. PCR was performed in a total volume of 25 μl, containing 1.5 μl of DNA as a template by mixing: sterile *dd*H_2_O (17.00 μl), 2 μl MgCl_2_ [2.5 mM], 2.5 μl 10X reaction buffer (10X NH_4_, Meridian Bioscience, Inc., Cincinnati, Ohio), 0.8 μl of deoxynucleotide triphosphates (dNTPs) [10 μM], 0.5 μl of each primer [10 μM], 0.20 μl of Taq DNA polymerase [1u/μl]. PCR was performed in a thermal cycler (Bio-Rad, C1000 Touch) using the following cycling protocol: 95 °C for 10 min (polymerase activation), followed by 31 cycles of 95 °C for 1 min (denaturation), 64° for 45 min (annealing), 72 °C for 1 min (extension), and 72 °C for 1 min (final extension). The PCR 700 bp long amplified product was visualized in 1.5% agarose gel.

PCR products were purified with SureClean Plus (Bioline, Meridian Bioscience) and sent to the sequencing provider (Sanger sequencing method; BMR Genomics s.r.l.). All the nucleotide sequences were screened with Chromas [38] and Mega 11 software [39]. Sequence species identity was confirmed in the GenBank^®^ database using nucleotide Basic Local Alignment Search Tool (*BLASTn)* [40].

### Thelazia callipaeda molecular detection

A subsample of flies was tested for the presence of *T*. *callipeada*. The *cox1* region (689 bp) was amplified using primers COI_F (5′-TGATTGGTGGTTTTGGTAA-3′) and COI_R (5′- ATAAGTACGAGTATCAATATC-3′) [41]. PCR was performed in a total volume of 25 μl, containing 2 μl of DNA as a template by mixing: sterile *dd*H_2_O (14.65 μl), 1.5 μl MgCl_2_ [2.5 mM], 2.5 μl 10X reaction buffer (10X NH_4_, Meridian Bioscience, Inc., Cincinnati, Ohio), 2.5 μl of dNTPs [10 μM], 0.8 μl of each primer [10 μM], 0.25 μl of Taq DNA polymerase [1u/μl]. PCR was performed in a thermal cycler (Bio-Rad, C1000 Touch) using the following cycling protocol: 95 °C for 10 min (polymerase activation), followed by 35 cycles of 95 °C for 1 min (denaturation), 55° for 30 min (annealing), 72 °C for 1 min (extension), and 72 °C for 1 min (final extension). The PCR 689 bp long amplified product was visualized in 1.5% agarose gel.

### Wolbachia endosymbiont molecular detection

The presence of the endosymbiont *Wolbachia* in the collected *Phortica* species was verified by PCR amplifying a portion of the *wsp* arthropod-specific gene (600 bp) [42]. The amplification reaction was carried out according to the following protocol (25 μl final volume): 2.5 μl 10X reaction buffer (10X ${NH}_{4}$, Meridian Bioscience, Inc., Cincinnati, Ohio), 2 μl MgCl2 [2.5 mM], 2.5 μl dNTPs [10 μM], 1 μl [10 μM] of primers 81F (5′-TGGTCCAATAAGTGATGAAGAAAC-3′) and 691R (5′-AAAAATTAAACGCTACTCCA-3′), 0.2 μl of Taq DNA polymerase [1u/μl], and 1 μl of DNA template. Amplification profile: 30 cycles at 94 °C for 30 min, 50 °C for 30 min, and 72 °C for 30 min. The amplified products were sequenced (Sanger sequencing method; BMR Genomics s.r.l.) and species identity was confirmed in the GenBank^®^ database using BLASTn [40].

### Statistical analysis

Different generalized additive models (hereafter named GAM-1; GAM-2; GAM-3; GAM-4) were fitted to determine the effect of the environmental parameters on the abundance of the *Phortica* spp. collected. The response variable was expressed as log-CPUE (the natural logarithm of Catch-Per-Unit-of-Effort, expressed as the number of collected flies/hour) to control for the variation in the sampling effort. The daily average of environmental data (temperature, humidity, barometric pressure, wind speed, and dew point) were retrieved from the nearest weather station (IMANZ6, 42.106° N, 12.078° E; located 2 km far from the sampling site; https://www.wunderground.com/). The three GAMs differ in the response variable. In GAM-1, only the log-CPUE for *P*. *oldenbergi* was considered, while in the other two models (GAM-2 and GAM-3) the response variable was the log-CPUE of *P*. *variegata* females (from fruit traps) and males (lacryphagous), respectively. Given the limited number of *P*. *semivirgo* and a single specimen of *P*. *okadai* collected during the whole sampling period, these species were excluded from the analysis. A selection of the predictors was carried out to avoid multicollinearity. In all the GAMs the parametric coefficients were sex, bait, and atmospheric pressure, while a smooth function was applied to windspeed, dew point, humidity, and temperature. A smooth function was also estimated for the date of collection (treated as day of the year) with an interaction between the factor variable bait (fruits/net). GAMs model structure was:

1 $${Y}_{i}= {\alpha + f}_{k}\left({X}_{ki}\right)+{\beta }_{j}\left({Z}_{ji}\right)+{\varepsilon }_{i}$$

where ${Y}_{i}$ is the log-CPUE, $\alpha$ is the intercept, $f$ is th *k*-th smooth term for the *k*-th non-linear predictor ($f$), $\beta$ is th *j*-th fixed effect for the *j*-th linear predictor ($Z$), and $\varepsilon$ is the error term for the *i*-th observation. To test if there was a significant difference in the mean number of *P*. *variegata* flies collected on fruit bait, another GAM (GAM-4) was fitted on the log-CPUE of flies collected with fruit bait. The residual analysis was performed in all the models and the root mean square error (RMSE) was computed to quantify the prediction accuracy (Supplementary information, Table S1). All statistical analyses were performed with R software (version 4.3.1) [43] and the packages: mgcv and tidygam [44, 45].

## Results

In total 5564 *Phortica *spp. flies were collected from 2018 to 2020 (*N* = 52 sampling days) in the area and morphologically identified. The most abundant species was *P*. *variegata* (90.8%), followed by *P*. *oldenbergi* (9.1%; Supplementary information, Fig. S2). A total of three specimens of *P*. *semivirgo* (0.05%) were collected in 2018 (*N* = 1, by human bait) and 2020 (*N* = 2, by fruit bait) while a single specimen of *P*. *okadai* was collected in 2018 (0.02%, by human bait). The numbers *P*. *variegata* and *P*. *oldenbergi* collected per month, year, and sampling bait are reported in Tables 1 and 2.

**Table 1:** Number of *Phortica variegata* collected during the sampling period

Year	Month	*N* fruit-bait (% males)	*N* human-bait (% males)	Total
2018	April	22 (13.6%)	–	
	May	262 (17.9%)	20 (100%)	
	June	89 (28.1%)	–	
	July	148 (43.9%)	8 (100%)	
	August	42 (40.5%)	2329 (100%)	
	September	94 (43.6%)	394 (100%)	
	October	1 (0%)	10 (100%)	3419
2019	April	5 (0%)	0	
	May	–	–	
	June	22 (27.3%)	11 (100%)	
	July	18 (5.6%)	749 (100%)	
	August	–	173 (100%)	
	September	3 (66.7%)	98 (100%)	1079
2020	May	2 (0%)	16 (100%)	
	June	12 (0%)	11 (100%)	
	July	26 (0%)	53 (100%)	
	August	41 (80.5%)	213 (99.5%)	
	September	5 (0%)	151 (99.3%)	
	October	–	26 (100%)	556
Total				5054

**Table 2:** Number of *Phortica oldenbergi* collected during the sampling period

Year	Month	*N* fruit-bait (% males)	*N* human-bait (% males)		Total
2018	April	20 (0%)	–		
	May	189 (4.8%)	–		
	June	24 (4.1%)	–		
	July	31 (22.6%)	–		
	August	9 (22.2%)	–		
	September	123 (4%)	1 (100%)		
	October	–	1 (100%)		398
2019	April	7 (0%)	–		
	May	2 (0%)	–		
	June	4 (0%)	1 (0%)		
	July	16 (0%)	1 (0%)		
	August	–	–		
	September	2 (0%)	1 (0%)		
	October	–	–		32
2020	May	10 (0%)	–		
	June	18 (0%)	–		
	July	32 (0%)	2 (100%)		
	August	7 (0%)	–		
	September	7 (0%)	–		
	October	–	–		76
Total					506

BLASTn sequence analysis of the *cox1* gene (*N* = 95) confirmed the morphological identification as *P*. *variegata* (*N* = 84; 99.7% of nucleotide identity (ni); accession number PP990198), *P*. *semivirgo* (*N* = 1; 99.1% ni; accession number PP990202) and *P*. *okadai* (*N* = 1, 99.8% ni; accession number PP860587). Sequence analysis of *P*. *oldenbergi* (*N* = 9; accession number PP838740; Supplementary information) showed a similar nucleotide identity with *P*. *variegata* (95.5% ni), *P*. *okadai* (95.1% ni), and *P*. *semivirgo* (94.7% ni) as sequences for this species were not available in GenBank. A total of 101 individuals (*N* = 74 *P*. *variegata*, *N*= 20 *P*. *oldenbergi*, *N* = 3 *P*. *semivirgo*, *N* = 1 *P*. *okadai*) were analyzed for the presence of *Wolbachia*. All *P*. *oldenbergi* and *P*. *okadai* specimens examined scored positive for a fragment of the expected size whereas *P*. *variegata* and *P*. *semivirgo* were negative (Supplementary information, Fig. S1). All sequences were identical and were confirmed as belonging to *Wolbachia* strain A (100% identity with *Wolbachia* endosymbiont (group A) of *Melieria omissa*; accession numbers PP930348 and PP930349).

*Thelazia callipaeda* DNA was detected in the *P*. *variegata* subsample (N = 699) showing different infection rates: 1.26% in 2018 (*N* = 352), 1% in 2019 (*N* = 100) and 2.02% in 2020 (*N* = 247), whereas any *P*. *oldenbergi*, *P*. *semivirgo*, and the single *P*. *okadai* were positive.

The sex ratio of both *P*. *variegata* and *P*. *oldenbergi* collected each year was unbalanced (Tables 1 and 2), The male:female ratio for *P*. *variegata* was 6.7:1 in 2018, 24.7:1 in 2019, and 9.1:1 in 2020, while for *P*. *oldenbergi*, this ratio was 0.07:1 in 2018, 0:1 in 2019, and 0.03:1 in 2020. The sex ratio of flies collected on human bait substantially differed from those gathered on fruit.

**Fig. 1:**
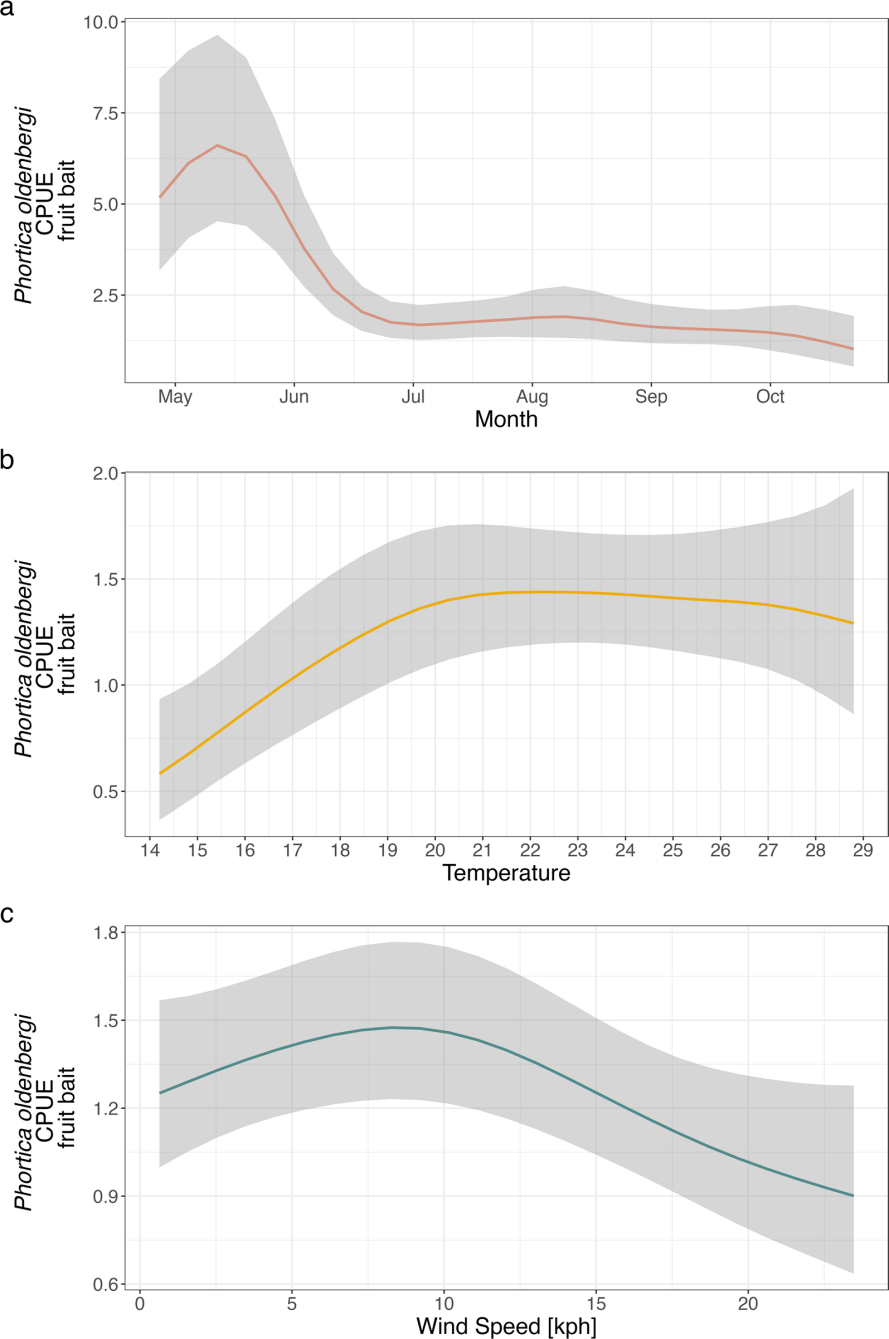
*Phortica oldenbergi* females’ population dynamics (GAM-1). Predicted log-CPUE of females collected with fruit bait in relation to the sampled months (**a**), temperature (**b**), and wind speed (**c**)

**Fig. 2:**
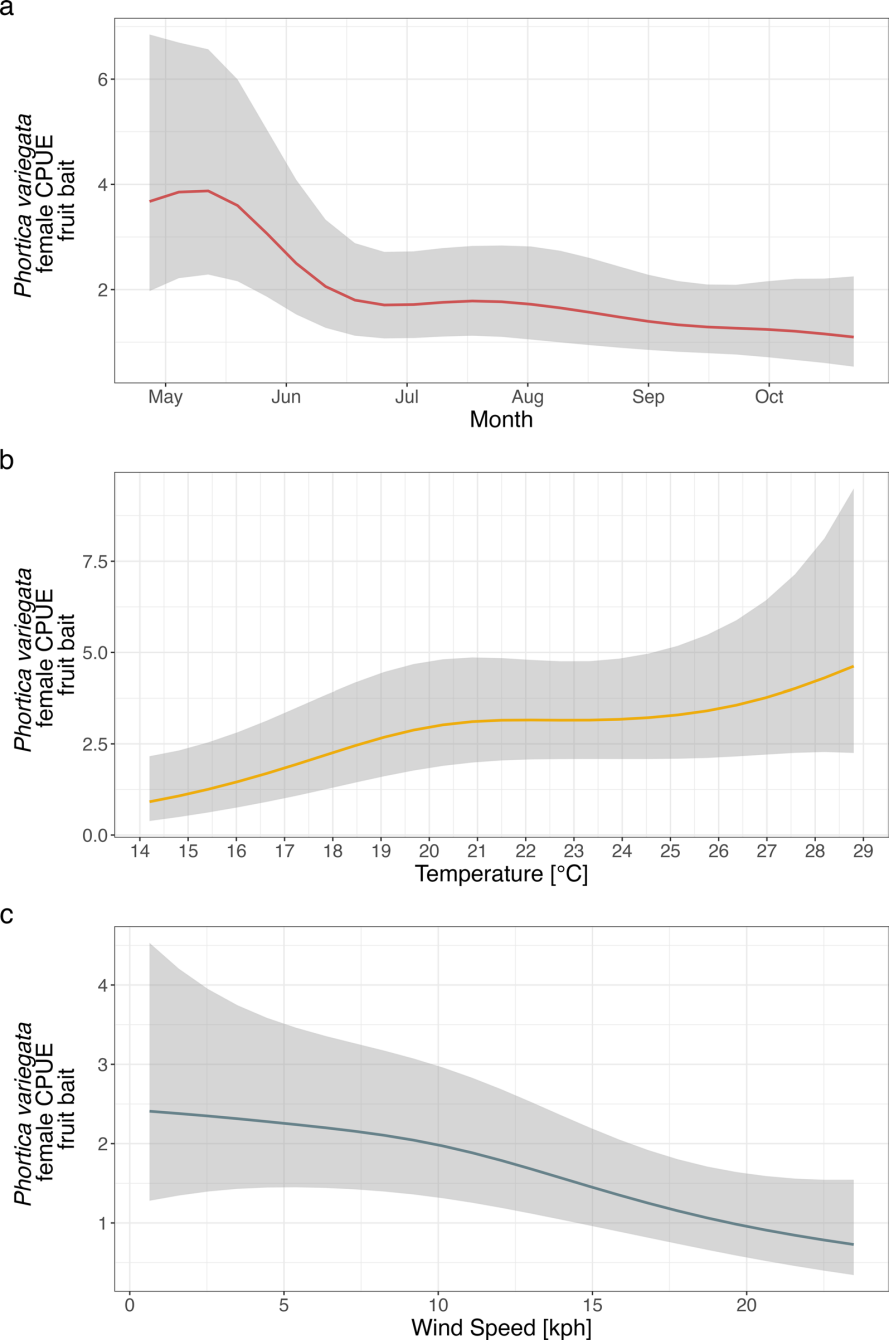
*Phortica variegata* females’ population dynamics (GAM-2). Predicted log-CPUE of females collected with fruit bait in relation to the sampled months (**a**), temperature (**b**), and wind speed (**c**)

**Fig. 3:**
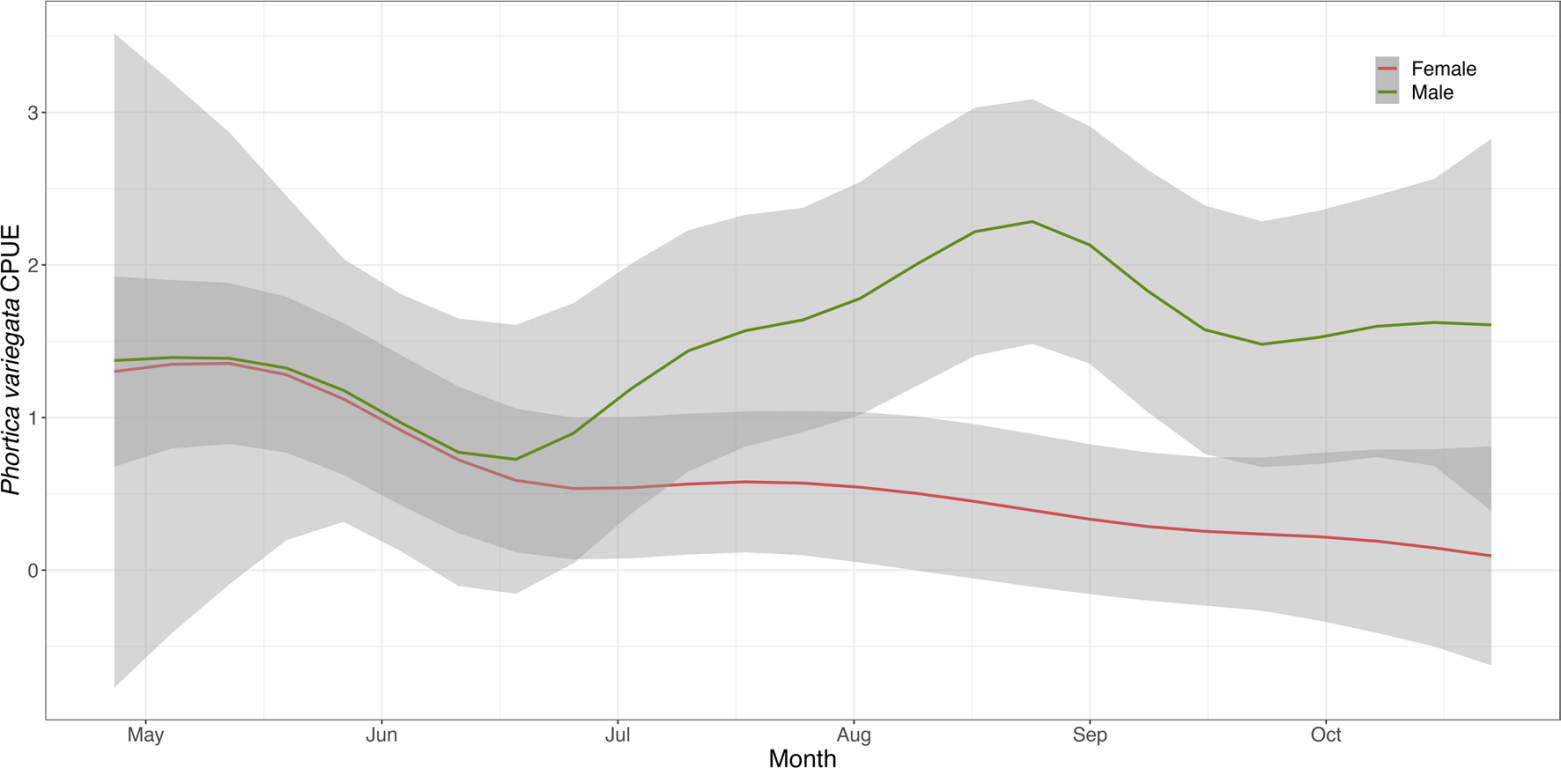
*Phortica variegata* males and females’ population dynamics (GAM-2 and GAM-3). Predicted log-CPUE for *P*. *variegata* males (green) collected with human bait and females (red) collected with fruit bait in the sampled months

**Fig. 4:**
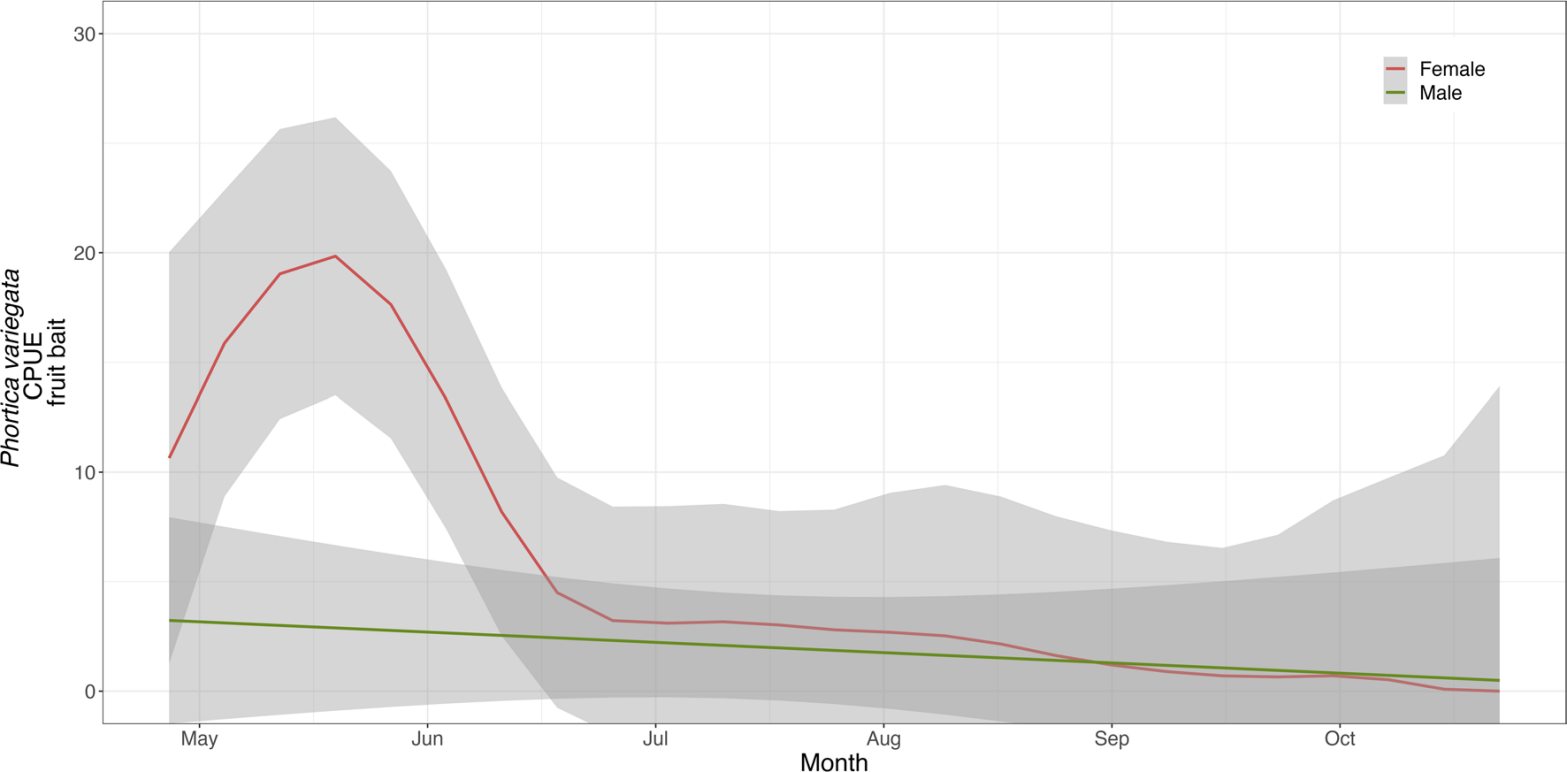
Fruit bait-collected *Phortica variegata* population dynamics (GAM-4). Predicted log-CPUE for fruits-collected *P*. *variegata* males (green) and females* (red) in the sampled months. *P-value 0.02

The results of the GAMs fitted on the log-CPUE of *Phortica* spp. showed a significant effect of the day of the year, temperature, and wind speed on the mean number of flies collected (Figs. 1 and 2). In all models the parametric coefficient showed a significant negative effect of barometric pressure (*P*-value < 0.01) and no significant effects of dew point and humidity. The seasonal dynamics of *P*. *oldenbergi* showed a first peak of abundance in May–June, followed by a decrease and a second (lower) peak of September–October [GAM-1; adjusted (adj.) *R*^2^ 0.46; Fig. 1a]. Similarly, *P*. *variegata* females were mainly abundant in the first part of the season (May–June) but showed a rapid decrease in June and a plateau until the end of sampling (GAM-2; adj. *R*^2^ 0.62; Fig. 2a). Conversely, numbers of *P*. *variegata* males (on human bait) rapidly increased in mid-June with a peak of abundance in August–September, then slightly decreased reaching a plateau until the end of sampling (GAM-3; adj. *R*^2^ 0.48; Fig. 3). Only *P*. *variegata* females collected on the fruit bait showed a significant trend (GAM-4; adj. *R*^2^ 0.30; Fig. 4). Single specimens of *P*. *oldenbergi* collected on human bait showed subequal number of males and females, with no seasonal trend detectable. Additionally, here the result is different from the sex ratio based mainly on the data from fruit bait.

## Discussion

This study showed for the first time the occurrence and seasonal dynamics of *Phortica* species living in sympatry within a forested area of Manziana (Central Italy), endemic for *T*. *callipeada*, in which *P*. *variegata* was the most abundant species (90% of the collected *Phortica* flies).

*Phortica oldenbergi* was collected throughout the sampling period in the study area, indicating, for the first time, its establishment in Central Italy, and a very surprising rediscovery this species in Europe. In fact, before this record, the species was documented in Europe, with only tree specimens of *P*. *oldenbergi* collected and described in Germany in the early twentieth century [24, 25]. Following this, six more female specimens were reported from Spain roughly 100 years later [26]. This led to the hypothesis that this species was accidentally introduced in Europe at the time [36] but could not settle in. Indeed, the species resembles the *Allophortica* subgenus, of which another four described species are known from the Afrotropical region [1]. The rare and patchy presence of this species in Europe might be explained by two hypotheses, both yet to be demonstrated: it might be a relict areal of its original distribution or it is the consequence of new introduction events. However, a recent study indicating this species as a competent vector of *T*. *callipeada* in laboratory conditions [20] highlights the need for further investigations to determine the vector capacity of this species in nature.

*Phortica semivirgo* was sporadically observed in Manziana, although it is considered a species native of Italian entomofauna [46]. The low numbers of *P*. *semivirgo* collected are consistent with available literature. This species was unknown to science until 1977, and only sporadically reported in Europe, except for Switzerland, where a trapping bottle system was used to collect the flies [47]. Nowadays, *P*. *semivirgo* seems more common, at least in central Europe, although the cases when it is collected in absence of *P*. *variegata* are rare [22, 48]. It is then possible that the abundance of this species was underestimated in this study owing to a less efficient trapping approach. Another possible explanation for the low density of *P*. *semivirgo* in the collection could be the different vertical micro-distribution in the canopy of *Phortica* spp. living in sympatric conditions, as indicated in literature [3, 48–50]; leading to the hypothesis that *P*. *semivirgo* may exploit upper levels of oak trees as a preferential habitat, while being less represented at ground level, where our sampling occurred.

Finally, *P*. *okadai*, a major vector of *T*. *callipeada* in the Asian region [2], was surprisingly found in the study area, representing the first record of this species in Europe. The finding of a single specimen of *P*. *okadai* suggests a possible accidental introduction by fruit trading. Further sampling in surrounding areas should be performed to increase information about the possible wider presence of *P*. *okadai* at least in Central Italy, which would be of paramount relevance for *T*. *callipeada* epidemiology, considering the major role of this vector in its original areal.

The oak forest of Manziana gave us the unique opportunity to study the population dynamics and feeding behavior of the taxa of the *Phortica* genus simultaneously. Population dynamics of all collected *Phortica* spp. showed a peak of abundance of females in May, possibly indicating a seasonal phase, when flies are focused on the feeding activity to store enough energy for reproduction after overwintering, as described in other Drosophilidae [51]. The sex ratio was highly unbalanced in the studied species. Most specimens of *P*. *variegata* were males collected with human bait, consistent with prior literature [8, 9, 29, 30, 47, 52], their numbers rapidly increasing after mid-June and peaking in late August (Fig. 3). Most *P*. *oldenbergi* flies collected were females caught with fruit bait, thus suggesting some connection to fermenting fruit, as observed in other *Phortica* species [52]. This does not necessarily mean an exclusive frugivory of the species, as a few specimens of both sexes were caught using human bait, indicating that lachryphagy can also occur. In addition, as observed in other *Drosophila* spp. [9] adult flies might also feed on microorganisms (e.g., yeasts) in tree sap or their larvae can possibly act as predators exploiting this biocenosis feeding on other insects. The low numbers of males collected in the study hampered the possibility to better clarify the feeding behavior of *P*. *oldenbergi* males. Similarly, during this sampling only a few specimens of *P*. *semivirgo* males were collected using fermenting fruit and human bait, yielding inconclusive results. Previous studies of various Steganinae flies have consistently reported a higher capture rate in traps baited with fermenting fruits near tree canopy [3, 53]. New sampling approaches applied to a different trophic niche, could be adopted in the future to further explore the population dynamics of *P*. *semivirgo*.

According to previous findings [9, 30, 52], and confirmed within this study, temperature constitutes a pivotal driver affecting the lachryphagous feeding behavior of *P*. *variegata* males. Furthermore, a significant effect of the temperature on the mean abundance of *Phortica* spp. on fruit bait was observed in this study, indicating its involvement also in this feeding activity. A plateau of the abundance of both fruit-baited *P*. *variegata* and *P*. *oldenbergi* was observed between 21 °C and 29 °C, suggesting that flies are less active during temperature peaks throughout the day (hottest days and hours of the year). This result was corroborated by the significant negative effect of the parametric coefficient of the pressure detected from all models. High atmospheric pressure results in the absence of cloud coverage and thus increase of temperatures with a consequent decrease in flies’ abundance. Our findings support a previous study on the flying activity of drosophilid flies, highlighting the general avoidance of several *Drosophila* spp. for higher temperatures and a species-specific thermopreferences affecting the range of total daily activity of these flies [54]. In addition, the models confirmed the negative effect of the wind speed on the mean abundance of collected flies, which can be reasonably explained by the higher energy required for flies for dispersal.

Regarding the positivity for *T*. *callipeada* of collected flies, *P*. *variegata* was the only species found infected, with rates ranging between 1% and 2.2%. Although both *P*. *semivirgo* and *P*. *oldenbergi* tested negative for the presence of *T*. *callipeada* during the study, considering their low sample size, it cannot be excluded that they could act as vectors in other areas with different ecological settings in which they may be more abundant. Nowadays, in Manziana the known hosts of *T*. *callipeada* are dogs, which are found infected during clinical screenings (R. P. Lia, unpublished data). On the basis of this evidence, *P*. *variegata* specimens might have been infected by feeding on neighboring dogs. Other information about potential sylvatic hosts is lacking, despite it being reasonable to suppose that other mammals, such as foxes, wild boars, beech martens, and squirrels, which are common in the area, might serve as reservoirs.

Finally, the exploratory investigation of arthropod-related *Wolbachia* strain in *Phortica* sample reveals an existing symbiosis in *P*. *oldenbergi* and *P*. *okadai*, while the symbiont appears to be absent in *P*. *variegata* and *P*. *semivirgo*. To the best of our knowledge, no *Phortica* species have previously been screened for the presence of *Wolbachia*, making this result the first indication of a symbiotic relationship within the *Phortica* genus. Given its presence in only some *Phortica* species, the most likely mechanism for the introduction of *Wolbachia* in *P*. *oldenbergi* and *P*. *okadai* involves horizontal transfer through shared environmental food sources [55]. In Diptera, one of the most notable effects of *Wolbachia* is cytoplasmic incompatibility, which enhances the bacterium’s spread by favoring the reproduction of infected females over uninfected ones. Thus, the presence of this symbiosis could potentially be considered among the factors influencing population dynamics and genetic diversity also in *Phortica* species [56].

## Conclusions

This study adds information on the ecological aspects of sympatric *Phortica* species within a forest of Central Italy, offering unprecedented insights into *P*. *variegata* and *P*. *oldenbergi* biology and ecology. The extensive collection and identification of these species underscore their seasonal variation of abundance and potential roles within the ecosystem, unravelling the complex interplay between environmental drivers, feeding activity, and vector behaviour, especially regarding the transmission of the eyeworm *T*. *callipeada*. In particular, the rediscovery and establishment of *P*. *oldenbergi* in Europe pose intriguing questions about its geographical origin, ecological role, and potential as a vector of *T*. *callipeada*, warranting further investigation. Similarly, the sporadic presence of *P*. *semivirgo* and the enigmatic observation of a single specimen of *P*. *okadai* in the Manziana area highlight the complexities of species establishment in new environments, possibly involving fruit trading. Further studies are imperative to explore the vectorial capacity of these species in natural settings, assess the impact of environmental changes on their populations, and develop strategies for monitoring and controlling their spread in relation to *T*. *callipeada* transmission to animals and humans.

## Supplementary Information

Additional file 1.

## Data Availability

No datasets were generated or analyzed during the current study.

## Abbreviations

GAM: Generalized additive model
CPUE: Catch-per-unit-of-effort
