# Supplementary material for: Population dynamics of sympatric Phortica spp. and first record of stable presence of Phortica oldenbergi in a Thelazia callipaeda-endemic area of Italy
Source: Parasit Vectors. 2024 Nov 6;17:455. doi: 10.1186/s13071-024-06526-9 (PMC11542218; doi:10.1186/s13071-024-06526-9)
Supplement: Supplementary file 1 — Additional file 1. [file 13071_2024_6526_MOESM1_ESM.docx]

**Population dynamics of sympatric *Phortica* spp. and first record of stable presence of *Phortica oldenbergi* in a *Thelazia callipaeda*-endemic area of Italy**

I. Bernardini^1,2¶^, C. Poggi^1¶^, D. Porretta^3^, J. Máca^4^, E. Perugini^1^, S. Manzi^1^, S. Gabrielli^1^, V. Pichler^1^, S. Latrofa^5^, J. Fourie^7^, R.P. Lia^5^, F. Beugnet^6^, D. Otranto^5^, M. Pombi^1*^

**Supplementary information**

**Table S1. Generalized additive models’ R-squared adjusted (R-sq adj.) and Root Mean Square errors (RMSE).**

| **Model** | **R-sq adj.** | **RMSE** |
| --- | --- | --- |
| GAM-1 | 0.46 | 0.57 |
| GAM-2 | 0.62 | 0.65 |
| GAM-3 | 0.48 | 1.03 |
| GAM-4 | 0.30 | 5.44 |

**Figure S1. *Wolbachia* molecular detection in *Phortica* species.**


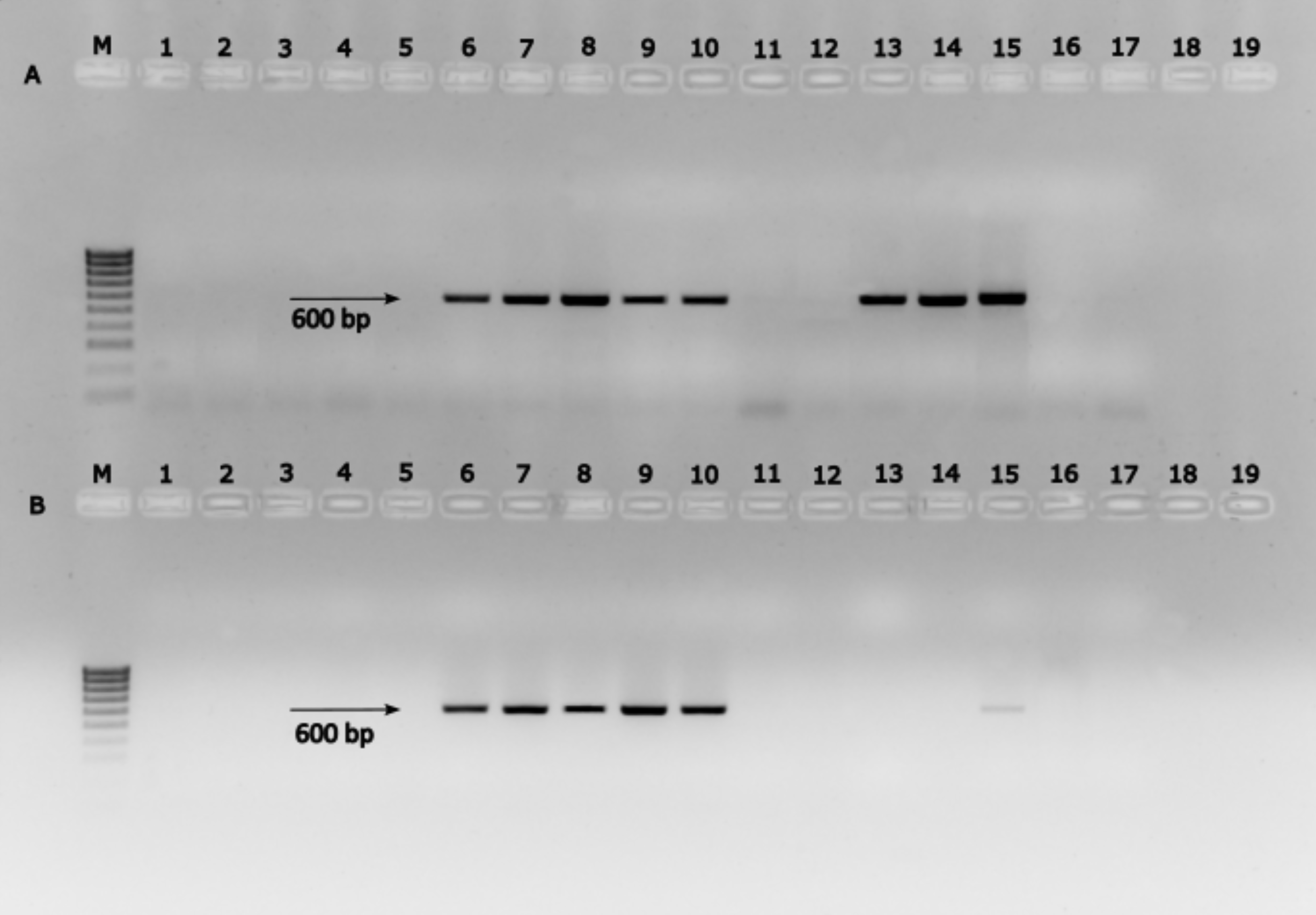


Results of PCR amplification of *wsp* gene fragment of *Wolbachia* strain A specific of the arthropods (600bp; Zhou et al. Proc R Soc Lond B Biol Sci. 1998. 265(1395):509–15). Each well corresponds to different specimens belonging to *Phortica* species tested. As positive controls have been taken mosquitoes belonging to *Culex pipiens* (1 male and 1 female), *Aedes albopictus* (1 male and 1 female) for which is known the presence of *Wolbachia*. As negative controls have been included *Thelazia callipaeda* (1 specimen), and *Dirofilaria immitis* (1 specimen), considering that the primers for *Wolbachia* strain A cannot amplify the filariae-specific *Wolbachia* strains.

Lane M: DNA 100bp marker

Rows A and B, lanes 1-5: *Phortica variegata*

Rows A and B, lanes 6-10: *Phortica oldenbergi*

Rows A and B, lanes 11-12; row B, lane 13: *Phortica semivirgo*

Row A, lane 13: *Phortica okadai*

Row A, lane 14: *Culex pipiens* female

Row B, lane 14: *Culex pipiens* male

Row A, lane 15: *Aedes albopictus* female

Row B, lane 15: *Aedes albopictus* male

Row A, lane 16: *Thelazia callipaeda*

Row B, lane 16: *Dirofilaria immitis*

Rows A and B, lanes 17-19: PCR negative controls

**Figure S2. *Phortica oldenbergi* morphological characters**


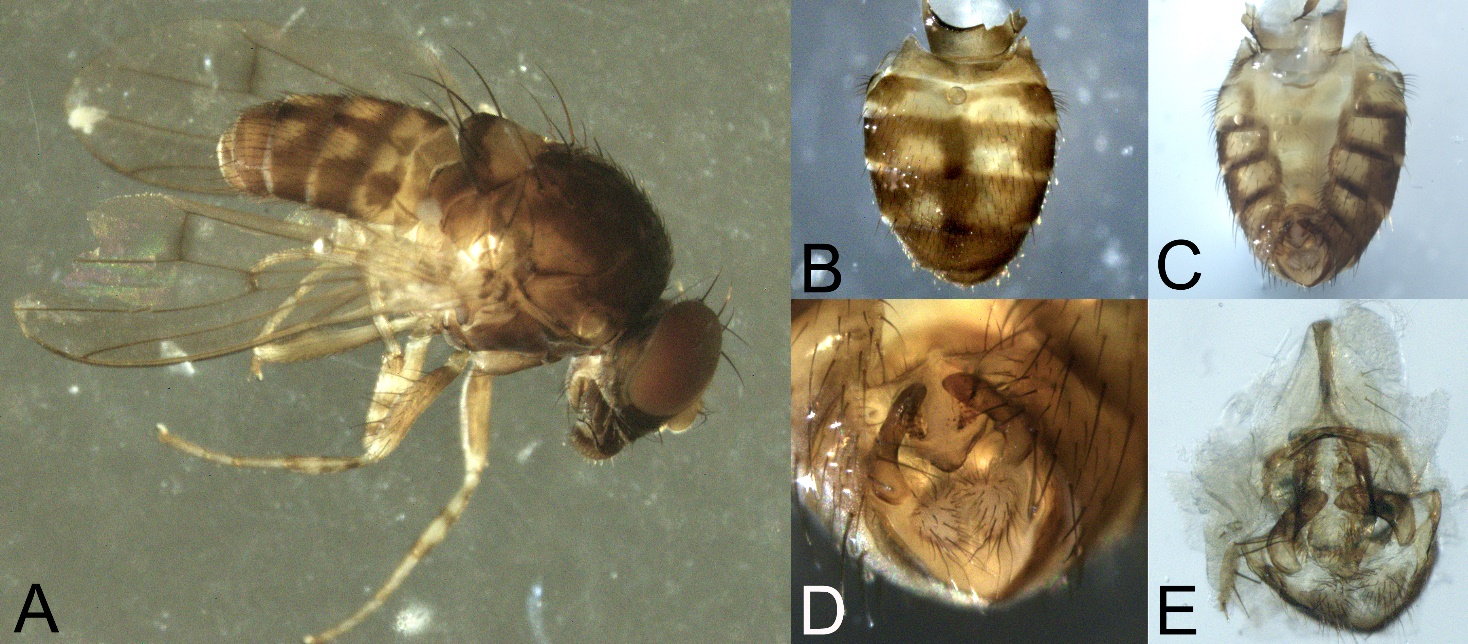


A: dorso-lateral view

B: dorsal view of the abdomen

C: ventral view of the abdomen

D: detail of the male copulatory organ

E: male terminalia dissected

According to the first description of *Phortica oldenbergi* by Duda, in this species tergites 2 to 5 are dark with two paramedial pale patches at the posterior margin and an additional pair of semilateral light patches behind the middle of the tergite (A, B). It has a supernumerary pair of scutellar bristles in the abdominal pattern (A), the loss of strong setae on the surstyli (D), and the parameres shape is markedly different from the other species collected in this area (D, E). The last males sternite is proximally divided into a bristle plate and has a V-shaped bare formation distally (C, D). The male genitalia (D, E) show minute periphallic organs and a seemingly bare clasper. The anterior parameres are tapering and apically bifid, covered by small nipples, with a laterally flattened apodeme (E).

***Phortica oldenbergi* sequence of the *cox1* fragment**

Amplified by primers UEA7-UEA10 (Lunt et al. Insect Mol Biol. 1996. 5(3):153–65). Accession number: PP838740 (458 bp)

>MA20-047

TTTTTATTCACTGTAGGAGGATTAACAGGAGTTGTATTASCTAATTCTTCAGTGGACATTATTTTACATGATACATATTATGTAGTTGCTCACTTTCACTATGKTTTATCTATAGGAGCTGTATTTGCTATTATAGCTRGATTTATTCACTGATACCCTTTATTTACKGGTTTAACTTTAAATTCAAAATGATTAAAAAGTCAATTTATTATTATATTTATKGGAGTAAATTTAACATTTTTCCCTCAACATTTTTTAGGATTASCTGGAATACCGCGACGTTATTCARATTACCCTGATGCTTATACTACTTGAAATGTAGTTTCAACAATCGGTTCATCAATTTCATTACTTGGAATTTTATTTTTCTTTTTTATTATTWGAGAAAGTTTAGTTTCTCAACGAGAAGTAATTTTTCCTATTCAATTAAATTCATCAATTGAATGATACCAAAATAC
